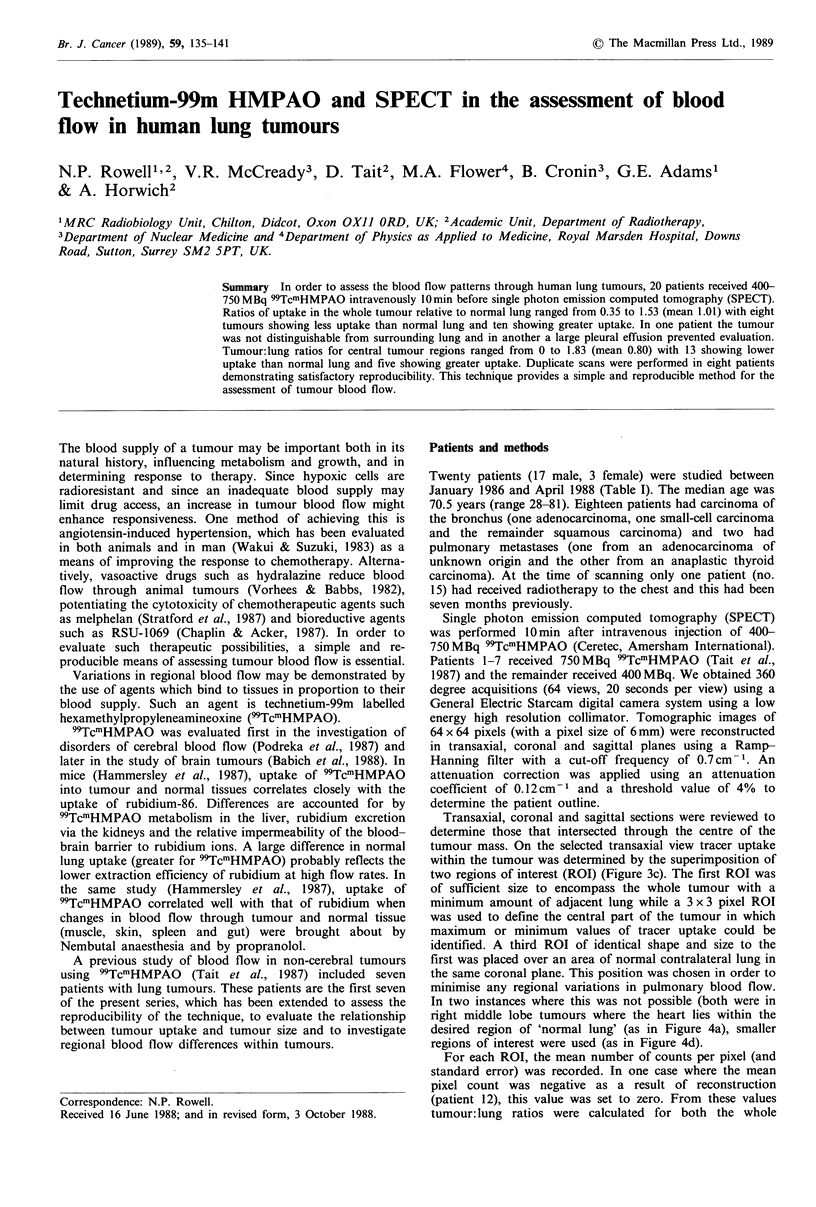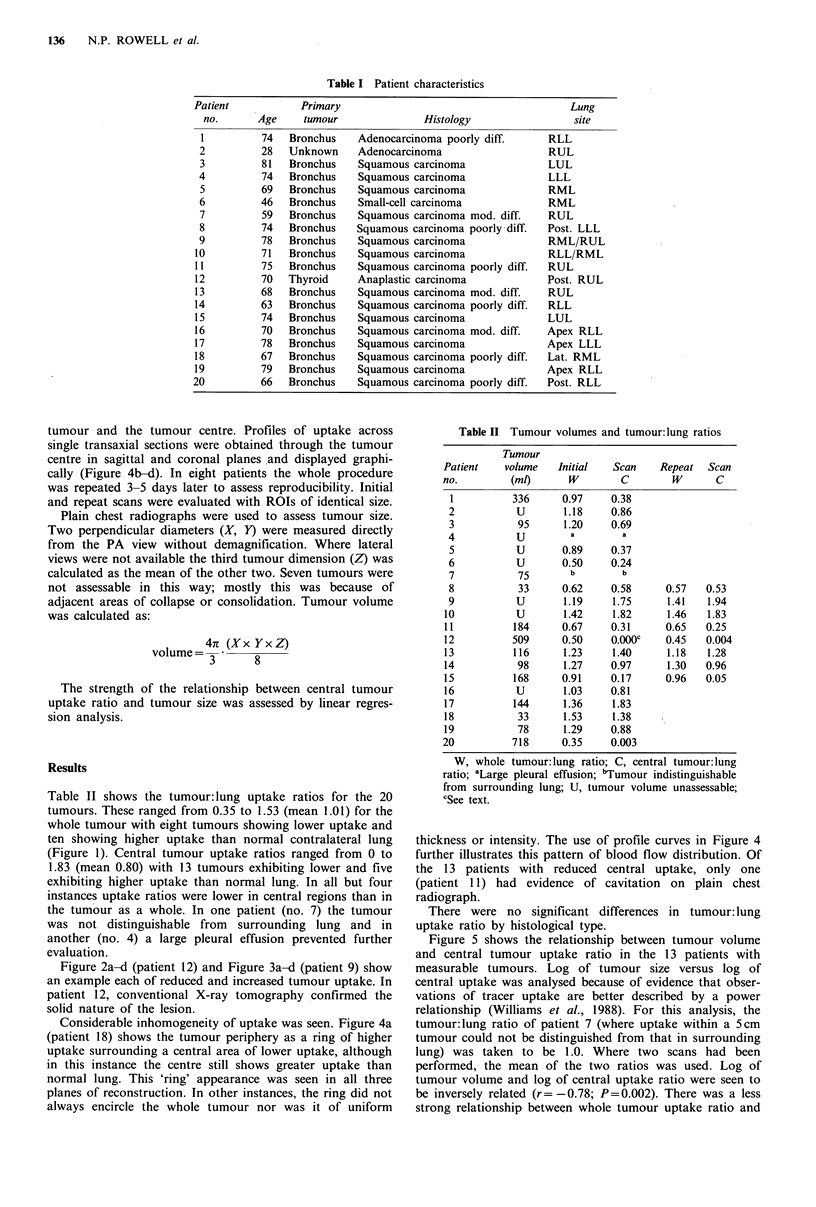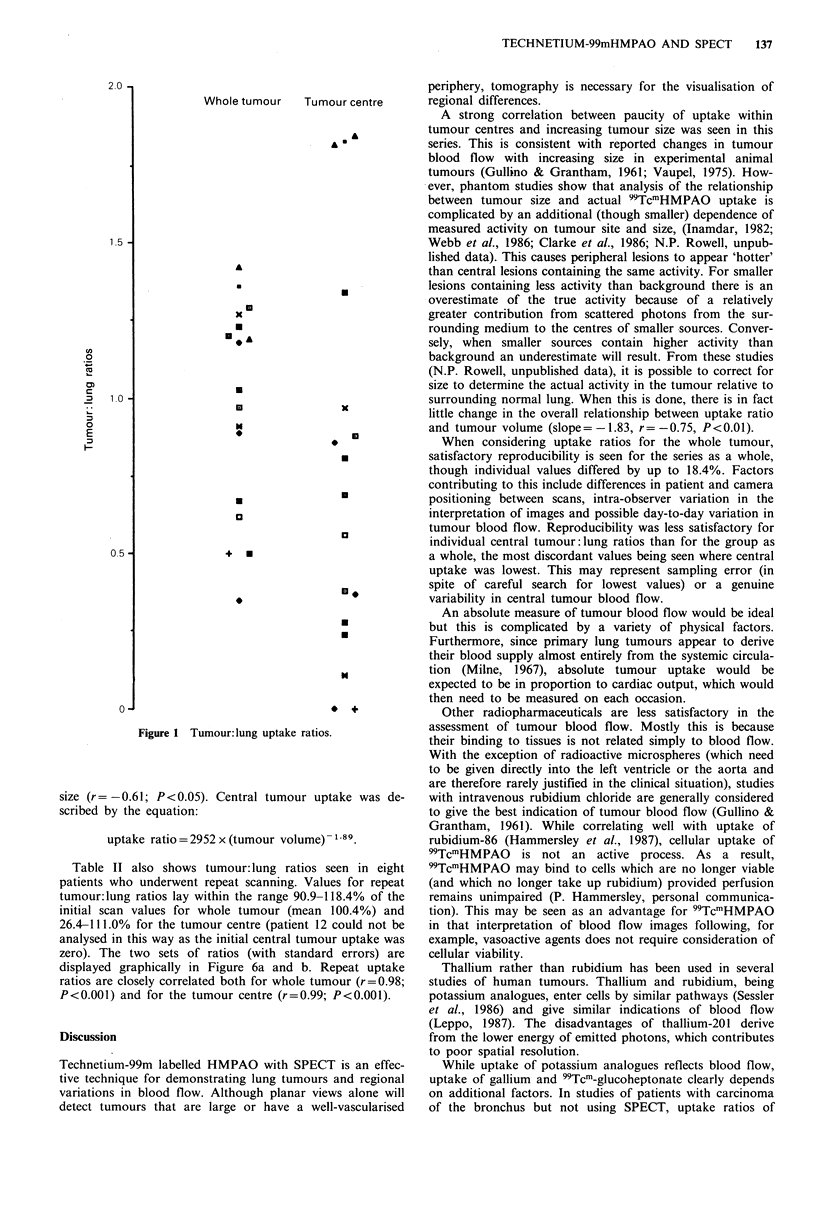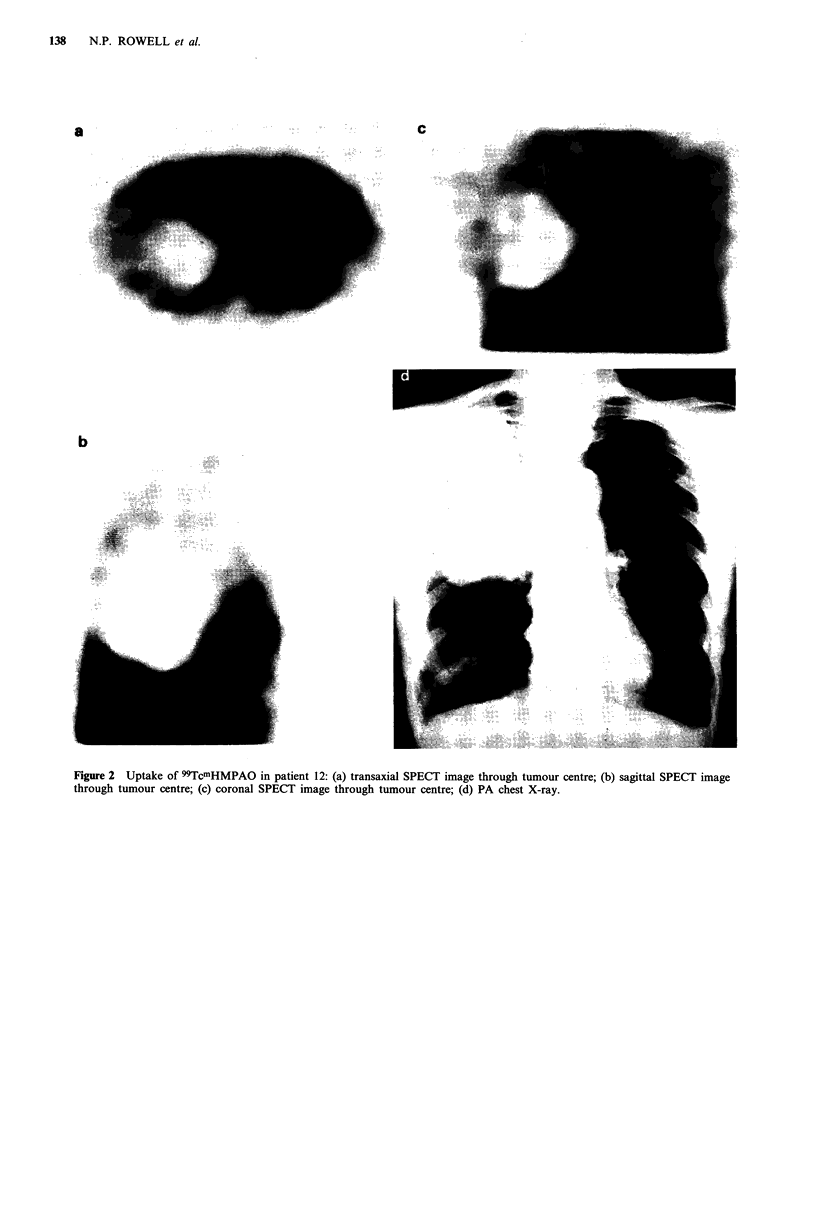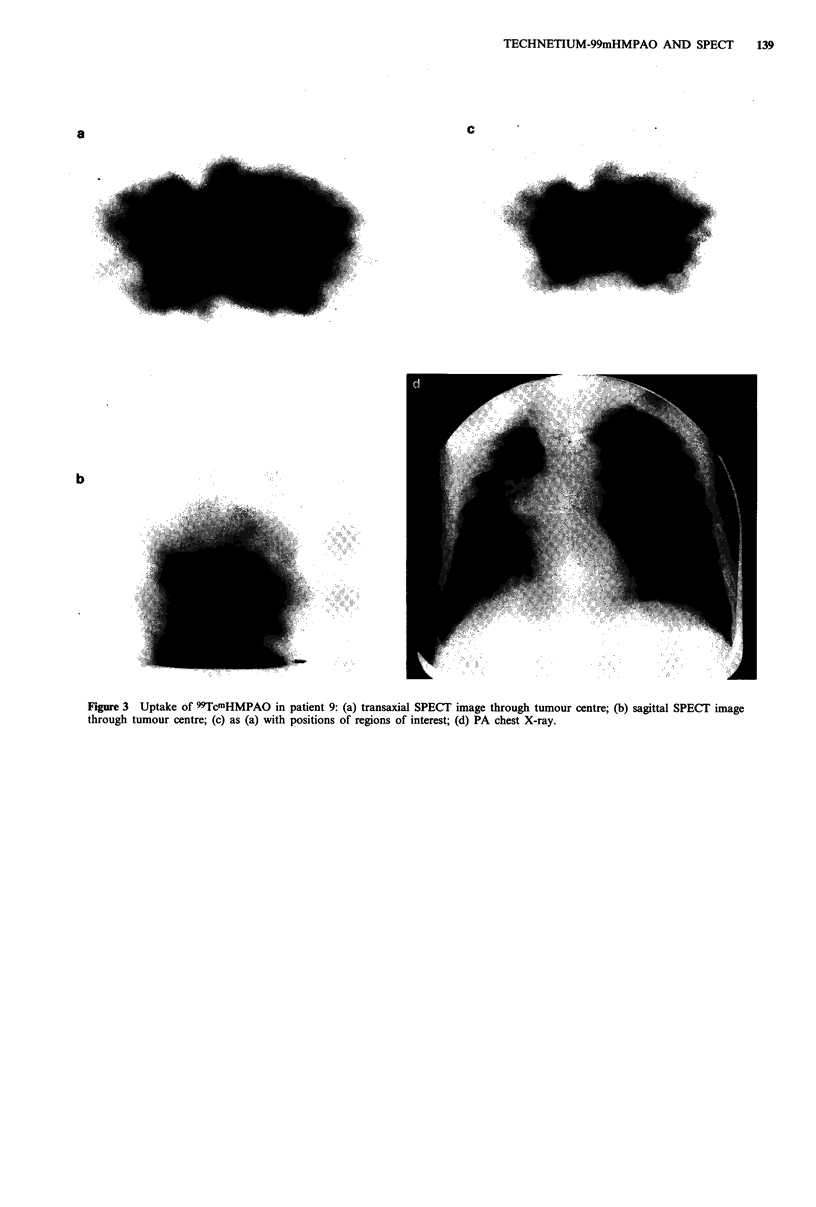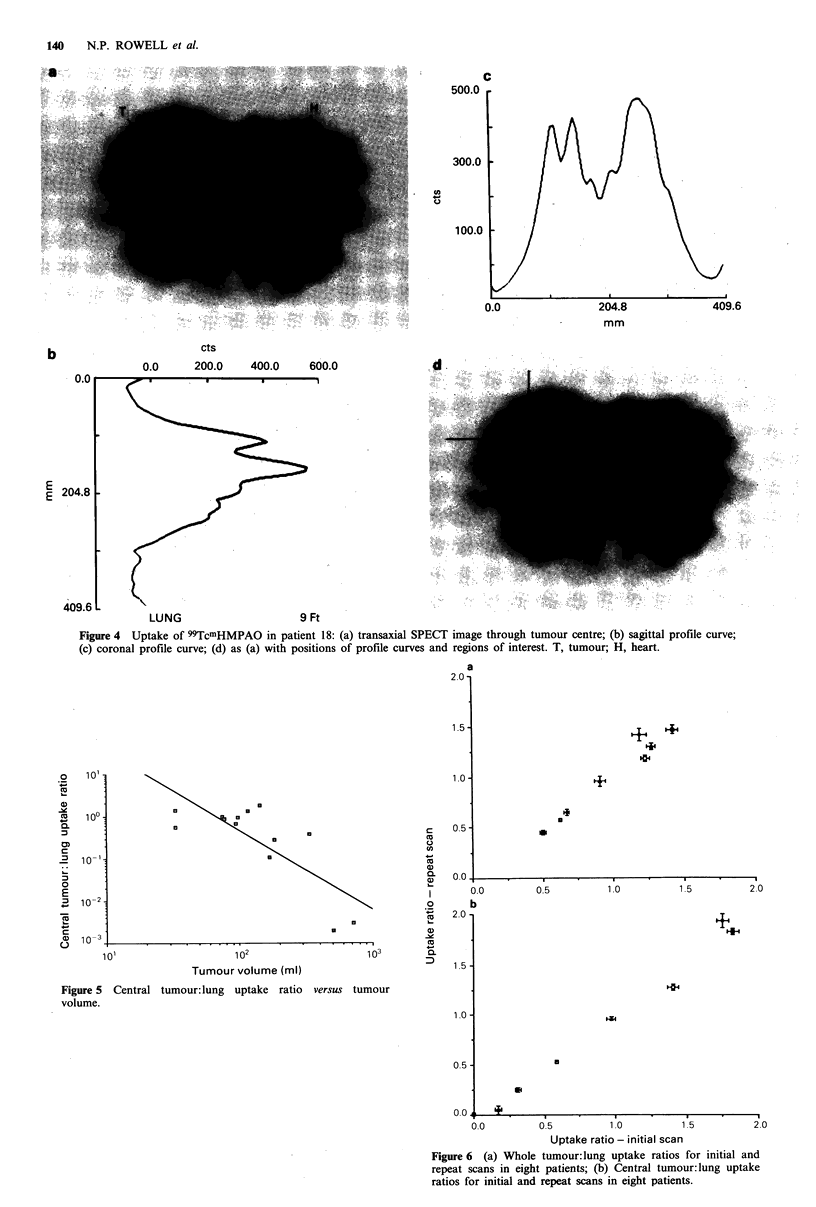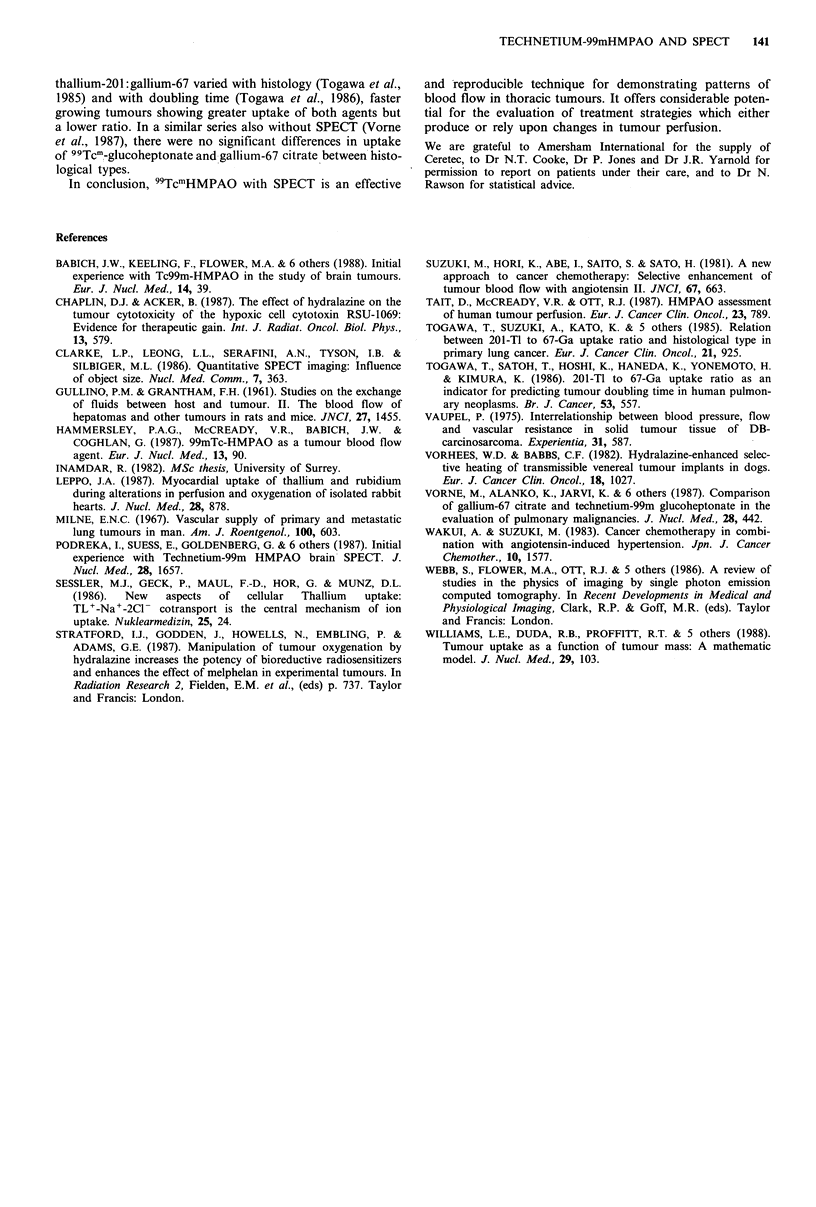# Technetium-99m HMPAO and SPECT in the assessment of blood flow in human lung tumours.

**DOI:** 10.1038/bjc.1989.27

**Published:** 1989-01

**Authors:** N. P. Rowell, V. R. McCready, D. Tait, M. A. Flower, B. Cronin, G. E. Adams, A. Horwich

**Affiliations:** MRC Radiobiology Unit, Didcot, Oxon, UK.

## Abstract

**Images:**


Br. J. Cancer (1989), 59, 135–141 © The Macmillan Press Ltd., 1989

### Technetium-99m HMPAO and SPECT in the assessment of blood flow in human lung tumours

N.P. Rowell[1,2], V.R. McCready[3], D. Tait[2], M.A. Flower[4], B. Cronin[3], G.E. Adams[1] & A. Horwich[2]

[1]*MRC Radiobiology Unit, Chilton, Didcot, Oxon OX11 0RD, UK;* [2]*Academic Unit, Department of Radiotherapy,* [3]*Department of Nuclear Medicine and* [4]*Department of Physics as Applied to Medicine, Royal Marsden Hospital, Downs Road, Sutton, Surrey SM2 5PT, UK.*

**Summary** In order to assess the blood flow patterns through human lung tumours, 20 patients received 400–750 MBq $^{99}Tc^{m}$HMPAO intravenously 10 min before single photon emission computed tomography (SPECT). Ratios of uptake in the whole tumour relative to normal lung ranged from 0.35 to 1.53 (mean 1.01) with eight tumours showing less uptake than normal lung and ten showing greater uptake. In one patient the tumour was not distinguishable from surrounding lung and in another a large pleural effusion prevented evaluation. Tumour:lung ratios for central tumour regions ranged from 0 to 1.83 (mean 0.80) with 13 showing lower uptake than normal lung and five showing greater uptake. Duplicate scans were performed in eight patients demonstrating satisfactory reproducibility. This technique provides a simple and reproducible method for the assessment of tumour blood flow.

The blood supply of a tumour may be important both in its natural history, influencing metabolism and growth, and in determining response to therapy. Since hypoxic cells are radioresistant and since an inadequate blood supply may limit drug access, an increase in tumour blood flow might enhance responsiveness. One method of achieving this is angiotensin-induced hypertension, which has been evaluated in both animals and in man (Wakui & Suzuki, 1983) as a means of improving the response to chemotherapy. Alternatively, vasoactive drugs such as hydralazine reduce blood flow through animal tumours (Vorhees & Babbs, 1982), potentiating the cytotoxicity of chemotherapeutic agents such as melphelan (Stratford *et al.*, 1987) and bioreductive agents such as RSU-1069 (Chaplin & Acker, 1987). In order to evaluate such therapeutic possibilities, a simple and reproducible means of assessing tumour blood flow is essential.

Variations in regional blood flow may be demonstrated by the use of agents which bind to tissues in proportion to their blood supply. Such an agent is technetium-99m labelled hexamethylpropyleneamineoxine ($^{99}Tc^{m}$HMPAO).

$^{99}Tc^{m}$HMPAO was evaluated first in the investigation of disorders of cerebral blood flow (Podreka *et al.*, 1987) and later in the study of brain tumours (Babich *et al.*, 1988). In mice (Hammersley *et al.*, 1987), uptake of $^{99}Tc^{m}$HMPAO into tumour and normal tissues correlates closely with the uptake of rubidium-86. Differences are accounted for by $^{99}Tc^{m}$HMPAO metabolism in the liver, rubidium excretion via the kidneys and the relative impermeability of the blood–brain barrier to rubidium ions. A large difference in normal lung uptake (greater for $^{99}Tc^{m}$HMPAO) probably reflects the lower extraction efficiency of rubidium at high flow rates. In the same study (Hammersley *et al.*, 1987), uptake of $^{99}Tc^{m}$HMPAO correlated well with that of rubidium when changes in blood flow through tumour and normal tissue (muscle, skin, spleen and gut) were brought about by Nembutal anaesthesia and by propranolol.

A previous study of blood flow in non-cerebral tumours using $^{99}Tc^{m}$HMPAO (Tait *et al.*, 1987) included seven patients with lung tumours. These patients are the first seven of the present series, which has been extended to assess the reproducibility of the technique, to evaluate the relationship between tumour uptake and tumour size and to investigate regional blood flow differences within tumours.

Correspondence: N.P. Rowell.
Received 16 June 1988; and in revised form, 3 October 1988.

### Patients and methods

Twenty patients (17 male, 3 female) were studied between January 1986 and April 1988 (Table I). The median age was 70.5 years (range 28–81). Eighteen patients had carcinoma of the bronchus (one adenocarcinoma, one small-cell carcinoma and the remainder squamous carcinoma) and two had pulmonary metastases (one from an adenocarcinoma of unknown origin and the other from an anaplastic thyroid carcinoma). At the time of scanning only one patient (no. 15) had received radiotherapy to the chest and this had been seven months previously.

Single photon emission computed tomography (SPECT) was performed 10 min after intravenous injection of 400–750 MBq $^{99}Tc^{m}$HMPAO (Ceretec, Amersham International). Patients 1–7 received 750 MBq $^{99}Tc^{m}$HMPAO (Tait *et al.*, 1987) and the remainder received 400 MBq. We obtained 360 degree acquisitions (64 views, 20 seconds per view) using a General Electric Starcam digital camera system using a low energy high resolution collimator. Tomographic images of $64 \times 64$ pixels (with a pixel size of 6 mm) were reconstructed in transaxial, coronal and sagittal planes using a Ramp–Hanning filter with a cut-off frequency of $0.7\,cm^{-1}$. An attenuation correction was applied using an attenuation coefficient of $0.12\,cm^{-1}$ and a threshold value of 4% to determine the patient outline.

Transaxial, coronal and sagittal sections were reviewed to determine those that intersected through the centre of the tumour mass. On the selected transaxial view tracer uptake within the tumour was determined by the superimposition of two regions of interest (ROI) (Figure 3c). The first ROI was of sufficient size to encompass the whole tumour with a minimum amount of adjacent lung while a $3 \times 3$ pixel ROI was used to define the central part of the tumour in which maximum or minimum values of tracer uptake could be identified. A third ROI of identical shape and size to the first was placed over an area of normal contralateral lung in the same coronal plane. This position was chosen in order to minimise any regional variations in pulmonary blood flow. In two instances where this was not possible (both were in right middle lobe tumours where the heart lies within the desired region of 'normal lung' (as in Figure 4a), smaller regions of interest were used (as in Figure 4d).

For each ROI, the mean number of counts per pixel (and standard error) was recorded. In one case where the mean pixel count was negative as a result of reconstruction (patient 12), this value was set to zero. From these values tumour:lung ratios were calculated for both the whole

**Table I** Patient characteristics

| *Patient no.* | *Age* | *Primary tumour* | *Histology* | *Lung site* |
|---|---|---|---|---|
| 1 | 74 | Bronchus | Adenocarcinoma poorly diff. | RLL |
| 2 | 28 | Unknown | Adenocarcinoma | RUL |
| 3 | 81 | Bronchus | Squamous carcinoma | LUL |
| 4 | 74 | Bronchus | Squamous carcinoma | LLL |
| 5 | 69 | Bronchus | Squamous carcinoma | RML |
| 6 | 46 | Bronchus | Small-cell carcinoma | RML |
| 7 | 59 | Bronchus | Squamous carcinoma mod. diff. | RUL |
| 8 | 74 | Bronchus | Squamous carcinoma poorly diff. | Post. LLL |
| 9 | 78 | Bronchus | Squamous carcinoma | RML/RUL |
| 10 | 71 | Bronchus | Squamous carcinoma | RLL/RML |
| 11 | 75 | Bronchus | Squamous carcinoma poorly diff. | RUL |
| 12 | 70 | Thyroid | Anaplastic carcinoma | Post. RUL |
| 13 | 68 | Bronchus | Squamous carcinoma mod. diff. | RUL |
| 14 | 63 | Bronchus | Squamous carcinoma poorly diff. | RLL |
| 15 | 74 | Bronchus | Squamous carcinoma | LUL |
| 16 | 70 | Bronchus | Squamous carcinoma mod. diff. | Apex RLL |
| 17 | 78 | Bronchus | Squamous carcinoma | Apex LLL |
| 18 | 67 | Bronchus | Squamous carcinoma poorly diff. | Lat. RML |
| 19 | 79 | Bronchus | Squamous carcinoma | Apex RLL |
| 20 | 66 | Bronchus | Squamous carcinoma poorly diff. | Post. RLL |

tumour and the tumour centre. Profiles of uptake across single transaxial sections were obtained through the tumour centre in sagittal and coronal planes and displayed graphically (Figure 4b–d). In eight patients the whole procedure was repeated 3–5 days later to assess reproducibility. Initial and repeat scans were evaluated with ROIs of identical size.

Plain chest radiographs were used to assess tumour size. Two perpendicular diameters ($X$, $Y$) were measured directly from the PA view without demagnification. Where lateral views were not available the third tumour dimension ($Z$) was calculated as the mean of the other two. Seven tumours were not assessable in this way; mostly this was because of adjacent areas of collapse or consolidation. Tumour volume was calculated as:

$$\text{volume} = \frac{4\pi}{3} \cdot \frac{(X \times Y \times Z)}{8}$$

The strength of the relationship between central tumour uptake ratio and tumour size was assessed by linear regression analysis.

### Results

Table II shows the tumour:lung uptake ratios for the 20 tumours. These ranged from 0.35 to 1.53 (mean 1.01) for the whole tumour with eight tumours showing lower uptake and ten showing higher uptake than normal contralateral lung (Figure 1). Central tumour uptake ratios ranged from 0 to 1.83 (mean 0.80) with 13 tumours exhibiting lower and five exhibiting higher uptake than normal lung. In all but four instances uptake ratios were lower in central regions than in the tumour as a whole. In one patient (no. 7) the tumour was not distinguishable from surrounding lung and in another (no. 4) a large pleural effusion prevented further evaluation.

Figure 2a–d (patient 12) and Figure 3a–d (patient 9) show an example each of reduced and increased tumour uptake. In patient 12, conventional X-ray tomography confirmed the solid nature of the lesion.

Considerable inhomogeneity of uptake was seen. Figure 4a (patient 18) shows the tumour periphery as a ring of higher uptake surrounding a central area of lower uptake, although in this instance the centre still shows greater uptake than normal lung. This 'ring' appearance was seen in all three planes of reconstruction. In other instances, the ring did not always encircle the whole tumour nor was it of uniform thickness or intensity. The use of profile curves in Figure 4 further illustrates this pattern of blood flow distribution. Of the 13 patients with reduced central uptake, only one (patient 11) had evidence of cavitation on plain chest radiograph.

There were no significant differences in tumour:lung uptake ratio by histological type.

Figure 5 shows the relationship between tumour volume and central tumour uptake ratio in the 13 patients with measurable tumours. Log of tumour size versus log of central uptake was analysed because of evidence that observations of tracer uptake are better described by a power relationship (Williams *et al.*, 1988). For this analysis, the tumour:lung ratio of patient 7 (where uptake within a 5 cm tumour could not be distinguished from that in surrounding lung) was taken to be 1.0. Where two scans had been performed, the mean of the two ratios was used. Log of tumour volume and log of central uptake ratio were seen to be inversely related ($r = -0.78$; $P = 0.002$). There was a less strong relationship between whole tumour uptake ratio and

**Table II** Tumour volumes and tumour:lung ratios

| *Patient no.* | *Tumour volume (ml)* | *Initial W* | *Scan C* | *Repeat W* | *Scan C* |
|---|---|---|---|---|---|
| 1 | 336 | 0.97 | 0.38 | | |
| 2 | U | 1.18 | 0.86 | | |
| 3 | 95 | 1.20 | 0.69 | | |
| 4 | U | [a] | [a] | | |
| 5 | U | 0.89 | 0.37 | | |
| 6 | U | 0.50 | 0.24 | | |
| 7 | 75 | [b] | [b] | | |
| 8 | 33 | 0.62 | 0.58 | 0.57 | 0.53 |
| 9 | U | 1.19 | 1.75 | 1.41 | 1.94 |
| 10 | U | 1.42 | 1.82 | 1.46 | 1.83 |
| 11 | 184 | 0.67 | 0.31 | 0.65 | 0.25 |
| 12 | 509 | 0.50 | 0.000[c] | 0.45 | 0.004 |
| 13 | 116 | 1.23 | 1.40 | 1.18 | 1.28 |
| 14 | 98 | 1.27 | 0.97 | 1.30 | 0.96 |
| 15 | 168 | 0.91 | 0.17 | 0.96 | 0.05 |
| 16 | U | 1.03 | 0.81 | | |
| 17 | 144 | 1.36 | 1.83 | | |
| 18 | 33 | 1.53 | 1.38 | | |
| 19 | 78 | 1.29 | 0.88 | | |
| 20 | 718 | 0.35 | 0.003 | | |

W, whole tumour:lung ratio; C, central tumour:lung ratio; [a]Large pleural effusion; [b]Tumour indistinguishable from surrounding lung; U, tumour volume unassessable; [c]See text.

**Figure 1** Tumour:lung uptake ratios.

size ($r = -0.61$; $P < 0.05$). Central tumour uptake was described by the equation:

$$\text{uptake ratio} = 2952 \times (\text{tumour volume})^{-1.89}.$$

Table II also shows tumour:lung ratios seen in eight patients who underwent repeat scanning. Values for repeat tumour:lung ratios lay within the range 90.9–118.4% of the initial scan values for whole tumour (mean 100.4%) and 26.4–111.0% for the tumour centre (patient 12 could not be analysed in this way as the initial central tumour uptake was zero). The two sets of ratios (with standard errors) are displayed graphically in Figure 6a and b. Repeat uptake ratios are closely correlated both for whole tumour ($r = 0.98$; $P < 0.001$) and for the tumour centre ($r = 0.99$; $P < 0.001$).

### Discussion

Technetium-99m labelled HMPAO with SPECT is an effective technique for demonstrating lung tumours and regional variations in blood flow. Although planar views alone will detect tumours that are large or have a well-vascularised periphery, tomography is necessary for the visualisation of regional differences.

A strong correlation between paucity of uptake within tumour centres and increasing tumour size was seen in this series. This is consistent with reported changes in tumour blood flow with increasing size in experimental animal tumours (Gullino & Grantham, 1961; Vaupel, 1975). However, phantom studies show that analysis of the relationship between tumour size and actual $^{99}Tc^{m}$HMPAO uptake is complicated by an additional (though smaller) dependence of measured activity on tumour site and size, (Inamdar, 1982; Webb *et al.*, 1986; Clarke *et al.*, 1986; N.P. Rowell, unpublished data). This causes peripheral lesions to appear 'hotter' than central lesions containing the same activity. For smaller lesions containing less activity than background there is an overestimate of the true activity because of a relatively greater contribution from scattered photons from the surrounding medium to the centres of smaller sources. Conversely, when smaller sources contain higher activity than background an underestimate will result. From these studies (N.P. Rowell, unpublished data), it is possible to correct for size to determine the actual activity in the tumour relative to surrounding normal lung. When this is done, there is in fact little change in the overall relationship between uptake ratio and tumour volume (slope $= -1.83$, $r = -0.75$, $P < 0.01$).

When considering uptake ratios for the whole tumour, satisfactory reproducibility is seen for the series as a whole, though individual values differed by up to 18.4%. Factors contributing to this include differences in patient and camera positioning between scans, intra-observer variation in the interpretation of images and possible day-to-day variation in tumour blood flow. Reproducibility was less satisfactory for individual central tumour:lung ratios than for the group as a whole, the most discordant values being seen where central uptake was lowest. This may represent sampling error (in spite of careful search for lowest values) or a genuine variability in central tumour blood flow.

An absolute measure of tumour blood flow would be ideal but this is complicated by a variety of physical factors. Furthermore, since primary lung tumours appear to derive their blood supply almost entirely from the systemic circulation (Milne, 1967), absolute tumour uptake would be expected to be in proportion to cardiac output, which would then need to be measured on each occasion.

Other radiopharmaceuticals are less satisfactory in the assessment of tumour blood flow. Mostly this is because their binding to tissues is not related simply to blood flow. With the exception of radioactive microspheres (which need to be given directly into the left ventricle or the aorta and are therefore rarely justified in the clinical situation), studies with intravenous rubidium chloride are generally considered to give the best indication of tumour blood flow (Gullino & Grantham, 1961). While correlating well with uptake of rubidium-86 (Hammersley *et al.*, 1987), cellular uptake of $^{99}Tc^{m}$HMPAO is not an active process. As a result, $^{99}Tc^{m}$HMPAO may bind to cells which are no longer viable (and which no longer take up rubidium) provided perfusion remains unimpaired (P. Hammersley, personal communication). This may be seen as an advantage for $^{99}Tc^{m}$HMPAO in that interpretation of blood flow images following, for example, vasoactive agents does not require consideration of cellular viability.

Thallium rather than rubidium has been used in several studies of human tumours. Thallium and rubidium, being potassium analogues, enter cells by similar pathways (Sessler *et al.*, 1986) and give similar indications of blood flow (Leppo, 1987). The disadvantages of thallium-201 derive from the lower energy of emitted photons, which contributes to poor spatial resolution.

While uptake of potassium analogues reflects blood flow, uptake of gallium and $^{99}Tc^{m}$-glucoheptonate clearly depends on additional factors. In studies of patients with carcinoma of the bronchus but not using SPECT, uptake ratios of

**Figure 2** Uptake of $^{99}Tc^{m}$HMPAO in patient 12: (a) transaxial SPECT image through tumour centre; (b) sagittal SPECT image through tumour centre; (c) coronal SPECT image through tumour centre; (d) PA chest X-ray.

**Figure 3** Uptake of $^{99}Tc^{m}$HMPAO in patient 9: (a) transaxial SPECT image through tumour centre; (b) sagittal SPECT image through tumour centre; (c) as (a) with positions of regions of interest; (d) PA chest X-ray.

**Figure 4** Uptake of $^{99}Tc^{m}$HMPAO in patient 18: (a) transaxial SPECT image through tumour centre; (b) sagittal profile curve; (c) coronal profile curve; (d) as (a) with positions of profile curves and regions of interest. T, tumour; H, heart.

**Figure 5** Central tumour:lung uptake ratio *versus* tumour volume.

**Figure 6** (a) Whole tumour:lung uptake ratios for initial and repeat scans in eight patients; (b) Central tumour:lung uptake ratios for initial and repeat scans in eight patients.

thallium-201:gallium-67 varied with histology (Togawa *et al.*, 1985) and with doubling time (Togawa *et al.*, 1986), faster growing tumours showing greater uptake of both agents but a lower ratio. In a similar series also without SPECT (Vorne *et al.*, 1987), there were no significant differences in uptake of $^{99}Tc^{m}$-glucoheptonate and gallium-67 citrate between histological types.

In conclusion, $^{99}Tc^{m}$HMPAO with SPECT is an effective and reproducible technique for demonstrating patterns of blood flow in thoracic tumours. It offers considerable potential for the evaluation of treatment strategies which either produce or rely upon changes in tumour perfusion.

We are grateful to Amersham International for the supply of Ceretec, to Dr N.T. Cooke, Dr P. Jones and Dr J.R. Yarnold for permission to report on patients under their care, and to Dr N. Rawson for statistical advice.

### References

BABICH, J.W., KEELING, F., FLOWER, M.A. & 6 others (1988). Initial experience with Tc99m-HMPAO in the study of brain tumours. *Eur. J. Nucl. Med.*, **14**, 39.

CHAPLIN, D.J. & ACKER, B. (1987). The effect of hydralazine on the tumour cytotoxicity of the hypoxic cell cytotoxin RSU-1069: Evidence for therapeutic gain. *Int. J. Radiat. Oncol. Biol. Phys.*, **13**, 579.

CLARKE, L.P., LEONG, L.L., SERAFINI, A.N., TYSON, I.B. & SILBIGER, M.L. (1986). Quantitative SPECT imaging: Influence of object size. *Nucl. Med. Comm.*, **7**, 363.

GULLINO, P.M. & GRANTHAM, F.H. (1961). Studies on the exchange of fluids between host and tumour. II. The blood flow of hepatomas and other tumours in rats and mice. *JNCI*, **27**, 1455.

HAMMERSLEY, P.A.G., McCREADY, V.R., BABICH, J.W. & COGHLAN, G. (1987). 99mTc-HMPAO as a tumour blood flow agent. *Eur. J. Nucl. Med.*, **13**, 90.

INAMDAR, R. (1982). *MSc thesis*, University of Surrey.

LEPPO, J.A. (1987). Myocardial uptake of thallium and rubidium during alterations in perfusion and oxygenation of isolated rabbit hearts. *J. Nucl. Med.*, **28**, 878.

MILNE, E.N.C. (1967). Vascular supply of primary and metastatic lung tumours in man. *Am. J. Roentgenol.*, **100**, 603.

PODREKA, I., SUESS, E., GOLDENBERG, G. & 6 others (1987). Initial experience with Technetium-99m HMPAO brain SPECT. *J. Nucl. Med.*, **28**, 1657.

SESSLER, M.J., GECK, P., MAUL, F.-D., HOR, G. & MUNZ, D.L. (1986). New aspects of cellular Thallium uptake: $TL^{+}$-$Na^{+}$-$2Cl^{-}$ cotransport is the central mechanism of ion uptake. *Nuklearmedizin*, **25**, 24.

STRATFORD, I.J., GODDEN, J., HOWELLS, N., EMBLING, P. & ADAMS, G.E. (1987). Manipulation of tumour oxygenation by hydralazine increases the potency of bioreductive radiosensitizers and enhances the effect of melphelan in experimental tumours. In *Radiation Research 2*, Fielden, E.M. *et al.*, (eds) p. 737. Taylor and Francis: London.

SUZUKI, M., HORI, K., ABE, I., SAITO, S. & SATO, H. (1981). A new approach to cancer chemotherapy: Selective enhancement of tumour blood flow with angiotensin II. *JNCI*, **67**, 663.

TAIT, D., McCREADY, V.R. & OTT, R.J. (1987). HMPAO assessment of human tumour perfusion. *Eur. J. Cancer Clin. Oncol.*, **23**, 789.

TOGAWA, T., SUZUKI, A., KATO, K. & 5 others (1985). Relation between 201-Tl to 67-Ga uptake ratio and histological type in primary lung cancer. *Eur. J. Cancer Clin. Oncol.*, **21**, 925.

TOGAWA, T., SATOH, T., HOSHI, K., HANEDA, K., YONEMOTO, H. & KIMURA, K. (1986). 201-Tl to 67-Ga uptake ratio as an indicator for predicting tumour doubling time in human pulmonary neoplasms. *Br. J. Cancer*, **53**, 557.

VAUPEL, P. (1975). Interrelationship between blood pressure, flow and vascular resistance in solid tumour tissue of DB-carcinosarcoma. *Experientia*, **31**, 587.

VORHEES, W.D. & BABBS, C.F. (1982). Hydralazine-enhanced selective heating of transmissible venereal tumour implants in dogs. *Eur. J. Cancer Clin. Oncol.*, **18**, 1027.

VORNE, M., ALANKO, K., JARVI, K. & 6 others (1987). Comparison of gallium-67 citrate and technetium-99m glucoheptonate in the evaluation of pulmonary malignancies. *J. Nucl. Med.*, **28**, 442.

WAKUI, A. & SUZUKI, M. (1983). Cancer chemotherapy in combination with angiotensin-induced hypertension. *Jpn. J. Cancer Chemother.*, **10**, 1577.

WEBB, S., FLOWER, M.A., OTT, R.J. & 5 others (1986). A review of studies in the physics of imaging by single photon emission computed tomography. In *Recent Developments in Medical and Physiological Imaging*, Clark, R.P. & Goff, M.R. (eds). Taylor and Francis: London.

WILLIAMS, L.E., DUDA, R.B., PROFFITT, R.T. & 5 others (1988). Tumour uptake as a function of tumour mass: A mathematic model. *J. Nucl. Med.*, **29**, 103.